# Radiotherapy and radio‐sensitization in *H3* ^K27M^ ‐mutated diffuse midline gliomas

**DOI:** 10.1111/cns.14225

**Published:** 2023-05-08

**Authors:** Chao Liu, Shuwen Kuang, Lei Wu, Quan Cheng, Xuan Gong, Jun Wu, Longbo Zhang

**Affiliations:** ^1^ Departments of Oncology Xiangya Hospital, Central South University Changsha China; ^2^ National Clinical Research Center for Geriatric Disorders Xiangya Hospital, Central South University Changsha China; ^3^ Department of Neurosurgery The Second Affiliated Hospital of Nanchang University Nanchang China; ^4^ Departments of Neurosurgery Xiangya Hospital, Central South University Changsha China; ^5^ Departments of Neurosurgery Yale School of Medicine New Haven Connecticut USA

**Keywords:** cell checkpoints, DMG, DNA damage and repair, H3K27M, radio‐sensitization

## Abstract

**Background:**

*H3* ^K27M^ mutated diffuse midline gliomas (DMGs) are extremely aggressive and the leading cause of cancer‐related deaths in pediatric brain tumors with 5‐year survival <1%. Radiotherapy is the only established adjuvant treatment of *H3* ^K27M^ DMGs; however, the radio‐resistance is commonly observed.

**Methods:**

We summarized current understandings of the molecular responses of *H3* ^K27M^ DMGs to radiotherapy and provide crucial insights into current advances in radiosensitivity enhancement.

**Results:**

Ionizing radiation (IR) can mainly inhibit tumor cell growth by inducing DNA damage regulated by the cell cycle checkpoints and DNA damage repair (DDR) system. In H3K27M DMGs, the aberrant genetic and epigenetic changes, stemness genotype, and epithelial‐mesenchymal transition (EMT) disrupt the cell cycle checkpoints and DDR system by altering the associated regulatory signaling pathways, which leads to the development of radio‐resistance.

**Conclusions:**

The advances in mechanisms of radio‐resistance in *H3* ^K27M^ DMGs promote the potential targets to enhance the sensitivity to radiotherapy.

## Graphical abstract

In *H3* ^K27M^ DMGs, radio‐resistance is commonly developed duo to cell‐intrinsic mechanisms such as the disruption of cell cycle checkpoints and DDR system and complex tumor microenvironment. In this review, we discussed the current understandings of the mechanisms of radio‐resistance in *H3* ^K27M^ DMGs and summarized the advances in radiosensitivity enhancement.

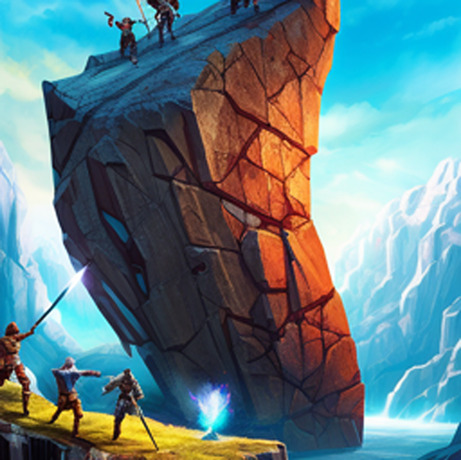

## 1 INTRODUCTION

Glioma is one of the most common brain malignancies. Although the integrative therapies including surgeries and chemoradiotherapies are applied in clinical treatment, the outcomes of glioma patients are unsatisfied.^1^, ^2^ Recent progress in glioma genomic sequencing suggests that somatic mutations are closely associated with the biology of tumors and the prognoses of patients, which results in the integration of both histopathology and molecular abnormalities in the WHO classification of central nervous system tumor in 2016.^3^

*H3* ^K27M^ mutations are prevalent in diffuse midline gliomas (DMGs) leading poor survival of less than 12 months^4^, ^5^, ^6^ (All of the abbreviations are listed in Table 1). Current clinical management of *H3* ^K27M^‐mutated DMGs include surgical resections and postoperative adjuvant therapies such as radiotherapy and chemotherapies.^7^ However, due to the infiltrative and diffuse biological behavior of *H3* ^K27M^‐mutated DMGs,^8^, ^9^ as well as the anatomical adjacent with most important brain structures such as thalamus, brainstem and medulla oblongata, the total resection of tumors is impractical.^8^, ^9^, ^10^, ^11^, ^12^ Thus, the adjuvant therapies are essential to prevent tumor recurrence and metastasis in *H3* ^K27M^ DMGs. Although the resistance of first‐line temozolomide has commonly observed in *H3* ^K27M^ DMGs due to the universal O6‐methylguanine‐DNA methyltransferase (MGMT) promotor unmethylation and intact blood–brain barrier (BBB).^13^, ^14^ Radiotherapy, as a standard and efficient strategy, can significantly improve the life qualities of *H3* ^K27M^ DMGs with 70%–80% of patients obtaining temporary symptom relief and increased survival.^15^, ^16^, ^17^, ^18^ Ionizing radiation (IR) can inhibit tumor growth by inducing DNA damage directly or through reactive oxygen species (ROS).^19^ The cell cycle checkpoints and DNA damage repair (DDR) system are essential for regulating the process.^20^ In *H3* ^K27M^ DMGs, the aberrant genetic and epigenetic changes, stemness genotype, and epithelial–mesenchymal transition (EMT) disrupt the cell cycle checkpoints and DDR system by altering the associated regulatory signaling pathways,^9^, ^21^, ^22^, ^23^ which could lead to the development of radio‐resistance. In clinical, a large proportion of *H3* ^K27M^ DMGs relapses within 7 months after radiotherapy.^4^, ^24^ To improve the sensitivity of tumor cells to radiation, extensive investigations have been conducted to understand the mechanisms of radio‐resistance and discover the approach to enhance the radiosensitivity including modulating radiotherapy doses and fractions and a combination of immunotherapy and chemotherapy. Here, we provide crucial insights into the molecular responses of *H3* ^K27M^ DMGs to radiotherapy and current advances in radiosensitivity enhancement.

**TABLE 1:** Abbreviations.

Abbreviations	Descriptions
ACVR1	Activin A receptor, type I
APE‐1	Apurinic/apyrimidinic endonuclease 1
ATM	Ataxia telangiectasia mutated
ATR	Ataxia telangiectasia and Rad3 related
ATRIP	ATR‐interacting protein
AURKA	Aurora kinase A
BBB	Brain blood barrier
BRD	Bromodomain protein
BMI1	B lymphoma moloney murine leukemia virus insertion region 1
BRCA1	Breast cancer type 1 susceptibility protein
BRCA2	Breast cancer type 2 susceptibility protein
CAK	CDK activated kinase
CDKs	Cyclin dependent kinases
CED	Convection‐enhanced delivery
CHKs	Checkpoint kinases
CKI	Cyclin‐dependent kinase inhibitor
CSCs	Cancer stem cells
CtIP	Ctbp‐interacting protein
CTV	Clinical tumor volume
DDR	DNA damage repair
DIPG	Diffuse intrinsic pontine glioma
DMGs	Diffuse midline gliomas
DNA pol	DNA polymerase
DNA‐PKcs	DNA‐dependent protein kinase catalytic subunit
DRD2	Dopamine receptor D2
E2F	Early 2 factor
EGFR	Epidermal growth factor receptor
EMT	Epithelial‐mesenchymal transition
ETAA1	ETAA1 activator of ATR kinase
EZH2	Zeste homolog 2
FANCD2	FA complementation group D2
FEN‐1	Free endonuclease 1
GTV	Gross tumor volume
HDAC	Histone deacetylases
HR	Homologous recombination
IR	Ionizing radiation
LigIII	Ligase III
LigIV	Ligase IV
MDC1	Mediator of DNA damage checkpoint 1
MGMT	O6‐methylguanine‐DNA methyltransferase
MRE11	Meiotic recombination 11 homolog 1
MRN	MRE11/RAD50/NBS1
NBS1	Phosphopeptide‐binding Nijmegen break‐age syndrome protein 1
NHEJ	Nonhomologous end‐joining
NQO1	NAD(P)H quinone oxidoreductase 1
PALB2	Partner and localizer of BRCA2
PARP1	Poly (ADP‐ribose) polymerase 1
PCNA	Proliferation cell nuclear antigen
PDGFRα	Platelet‐derived growth factor receptor α
PEK‐1	Poly nucleotide kinase 1
PLK1i	Polo‐like kinase 1 inhibitor
PNK	Poly nucleotide kinase
polyB	DNA polymerase
PPM1D	Protein phosphatase magnesium‐dependent 1 delta
PRC1	Polycomb repressive complex 1
PRC2	Polycomb repressive complex 2
PTIP	Pax transactivation domain interacting protein
PTMs	Post‐translational modifications
RAD50	ATP‐binding cassette (ABC)‐ATPase
RAD51	RAD51 recombinase
RB	Retinoblastoma
RECQ4	RecQ helicase
RFC	Replication factor C
RIF1	Replication timing regulatory factor 1
RNF8	RING finger protein 8
ROS	Reactive oxygen species
RT	Radiation therapy
RTKs	Receptor tyrosine kinases
SHH	Sonic hedgehog
SSBR	Single‐strand breaks repair
ssDNA	Single strand DNA
TMZ	Temozolomide
TOPBP1	DNA topoisomerase 2‐binding protein 1
WRN	Werner syndrome helicase
XRCC1	X‐ray repair cross complementing protein1
XRCC4	X‐ray repair cross complementing protein 4
γH2AX	Histone H2A.X
53BP1	P53 binding protein1

## 2 ONCOGENIC MECHANISMS OF *H3* ^K27M^ MUTATION

### 2.1 Histones and cancers

Histones including four core proteins H3, H4, H2A and H2B are the major structural components of chromatins which play important roles in gene expression and epigenetic modification.^25^, ^26^ The protruding N‐terminal amino acid tail of a histone is subject to various post‐translational modifications (PTMs) such as methylation, acetylation, phosphorylation, ubiquitylation, and SUMOylation.^27^, ^28^, ^29^, ^30^, ^31^ Histone PTMs can active or decrease gene transcription through a local structural alteration of chromatin conducted by the interactions between histones and DNA.^32^ The alterations of histones are demonstrated to be associated with various cancers such as gliomas, sarcomas, head and neck cancers and carcinosarcomas.^33^, ^34^ Of note, “oncohistones”, the cancer‐associated histone mutations, are highly specific in certain type of cancers. For instance, *H3* ^K27M^ and *H3* ^G34R/V^ mutations are specifically detected in brain tumors, *H3* ^K36M^ and *H3* ^G34W/L^ are mostly detected in bone cancers, while *H1* mutations are commonly found in lymphomas.^35^

### 2.2 H3 mutations in DMGs

The mutations in the histone *H3* family including canonical *H3.1* (*H3C1*) and variant *H3.3* (*H3F3A*) dominantly occur in approximately 50% of DMGs and 80% of diffuse intrinsic pontine glioma (DIPG) with lysine to methionine substitution at position 27.^4^, ^15^, ^36^ However, *H3.3* and *H3.1* mutations differ in their pathological and molecular features, as well as clinical prognoses. Unlike the *H3.1*‐mutant tumors are usually restrictedly located in pons, the *H3.3*‐mutant tumors can occur along the brain midline.^37^, ^38^ In addition, *H3.3* alteration often co‐occur with *TP53* mutation and platelet‐derived growth factor receptor α (*PDGFRα*) upregulation, while activin A receptor, type I (*ACVR1*) mutation is detected only in *H3.1*‐mutant tumors.^37^, ^39^, ^40^ Moreover, *H3.3* mutation is associated with more aggressive tumor with early relapse and frequent metastases, and exhibit poor response to radiotherapy.^37^, ^41^, ^42^

### 2.3 Molecular mechanisms and signaling transduction in *H3^K27M^* tumor

**FIGURE 1:**
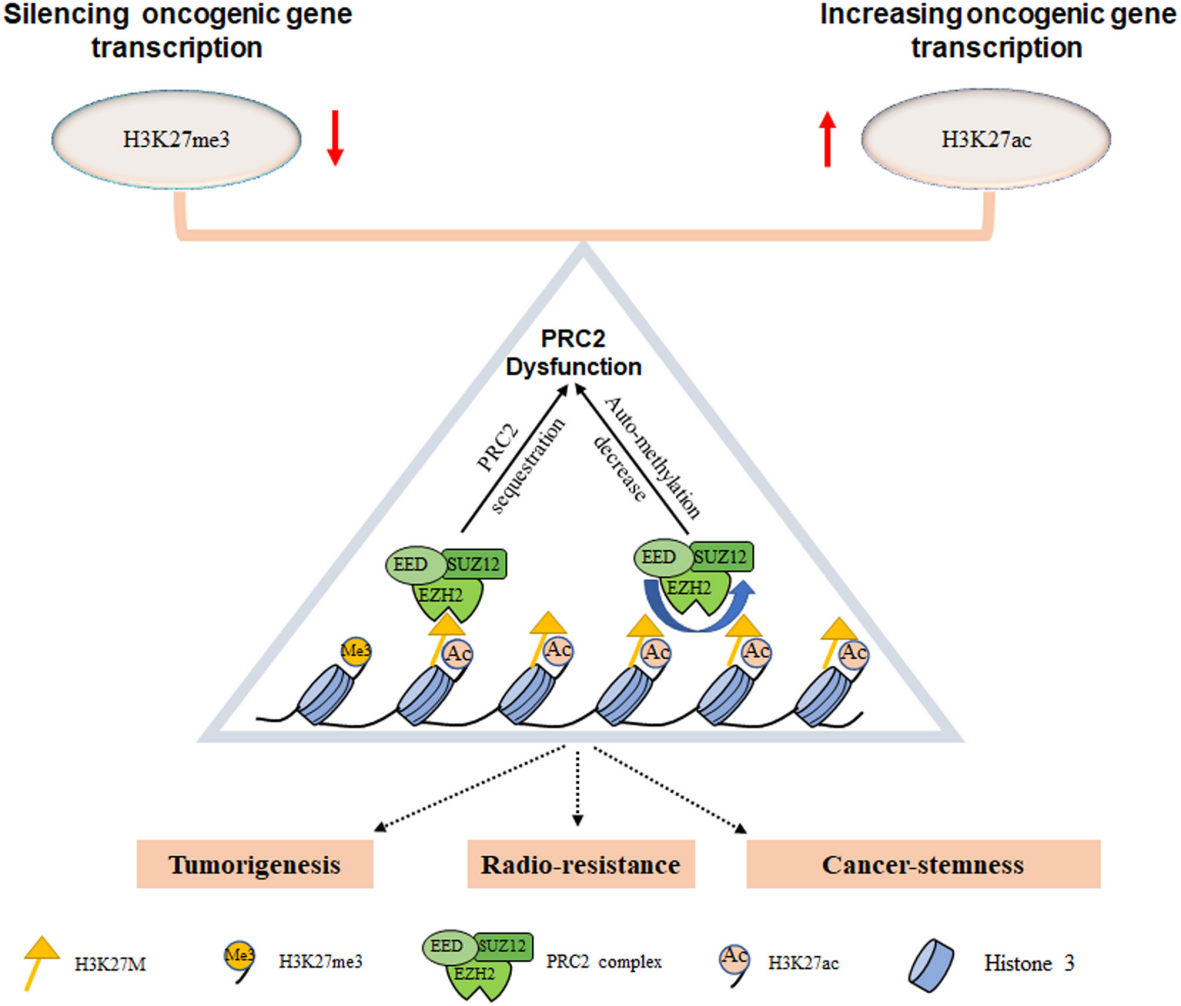
Diagram illustrating the epigenetic changes induced by *H3* ^K27M^ in DMGs.

In DMGs, *H3* ^K27M^ mutation can induce a global reduction of H3K27me3, an epigenetic mark to H3 indicating the trimethylation of lysine 27 on histone H3 protein, which is associated with transcriptional silencing of the key genes regulating cell differentiation via the formation of heterochromatic regions.^43^, ^44^, ^45^ H3K27me3 is catalyzed by the histone methyltransferase enhancer of zeste homolog 2 (EZH2), a subunit of polycomb repressive complex 2 (PRC2), which is the central modulator of H3K27me3‐decorated facultative heterochromatin.^46^, ^47^ The crystal structure analysis suggests that K27M‐mutant H3 can bind directly to the active SET domain of EZH2 with 16‐fold higher affinity than wildtype H3 leading to the sequestration of EZH2 and further impairment of PRC2 methyltransferase activity and the reduction of H3K27me3.^4^, ^15^, ^48^, ^49^ In addition, *H3* ^K27M^ mutation can decrease the auto‐methylation of EZH2/PRC2 which is required for histone methyltransferase activity, resulting the detention of H3K27me2 to H3K27me3.^50^, ^51^ Another epigenetic mark to H3 in *H3* ^K27M^ mutation cells is that the increased acetylation of H3K27 (H3K27ac), which is considered to be a super‐enhancer to promote gene transcription involved in stem cell differentiation and tumorigenesis^45^, ^52^ (Figure 1).

Beyond epigenetic signatures, the *H3* ^K27M^ mutation has been demonstrated to be associated with aberrant amplifications and overexpression of receptor tyrosine kinases (RTKs), a group of membrane proteins such as epidermal growth factor receptor (EGFR) and platelet‐derived growth factor receptor α (PDGFRα) regulating a set of biological functions including cell growth, survival, differentiation and migration.^53^, ^54^, ^55^, ^56^, ^57^ Overexpression of PDGFRα is commonly found in aggressive glioma tumors exhibiting upregulated proliferation and migration of cancer cells, and significantly associated to poor survival in *H3* ^K27M^ DMGs.^38^, ^54^, ^57^, ^58^, ^59^ In addition, *ACVR1* mutation, for example, is prevalent in *H3.1* ^K27M^ tumor which could upregulate SMAD1/5/8 signaling leading to cancer cell proliferation.^23^, ^40^, ^60^, ^61^, ^62^ PI3K/AKT/mTOR and MEK/ERK pathway, as downstream signaling of RTKs, are commonly activated in *H3* ^K27M^ mutation DMGs22,^9^, ^22^, ^63^ which contribute to tumorigenesis, metastasis, angiogenesis and therapy resistance resulting in poor clinical prognosis.^64^, ^65^, ^66^, ^67^, ^68^, ^69^

## 3 RADIOTHERAPIES AND MECHANISMS

Radiotherapy serves as cornerstone in the adjuvant treatment of *H3* ^K27M^ DMGs, which significantly improve the clinical outcomes in 70%–80% of patients.^16^, ^17^, ^18^ Currently, the recommend total dose setting of radiotherapy is 54–60 Gy with a daily fraction of 1.8–2.0 Gy over 6 weeks.^70^ Mechanically, radiation mainly inhibits tumor growth by inducing DNA damage.^19^ The damaged DNA would trigger tumor cell death once failure in the repairment. However, DNA damage detection and repair are complicated regulated by intricate intracellular and extracellular networks. Under certain circumstances, tumor cells can exhibit radio‐resistance due to cell‐intrinsic mechanisms and microenvironment resulting treatment failure and recurrence.^71^, ^72^ Thus, understanding the mechanisms underlying radio‐resistance and developing mechanism‐based radio sensitization to enhance radiotherapy responses would improve the outcomes of glioma patients. A set of sophisticated and highly regulated cellular processes can be triggered by the exposure of radiation. Among these, the cell cycle checkpoints and DDR system are the main components coordinated by complex signal transduction.^73^, ^74^ Mechanically, the sensors recognizing the radiation induced DNA damage can initiate the cascade activation of a series of kinases involved in the cell cycle checkpoints and DDR networks to complete DNA repairs.

### 3.1 Cell cycle checkpoints

Cell cycle checkpoints consisting of G1, intra‐S, G2 and spindle phase act as surveillance mechanisms and regulators to monitor the major events among the process of cell cycle (such as DNA damage). Once aberrant events are detected by the checkpoints during segregation at mitosis, a pulse would be placed to arrest the cell cycle which allows to provide a time for DNA repair in order to accurately passage bioinformation into daughter cells and maintain the genomic stability. For example, G1 phase checkpoint assure the damaged DNA cannot enter S phase, and G2 checkpoint could prevent the passing of abnormal DNA to future generations.^75^ The dysfunction of cell cycle checkpoints or failure in the repairment would trigger cell death eventually.

**FIGURE 2:**
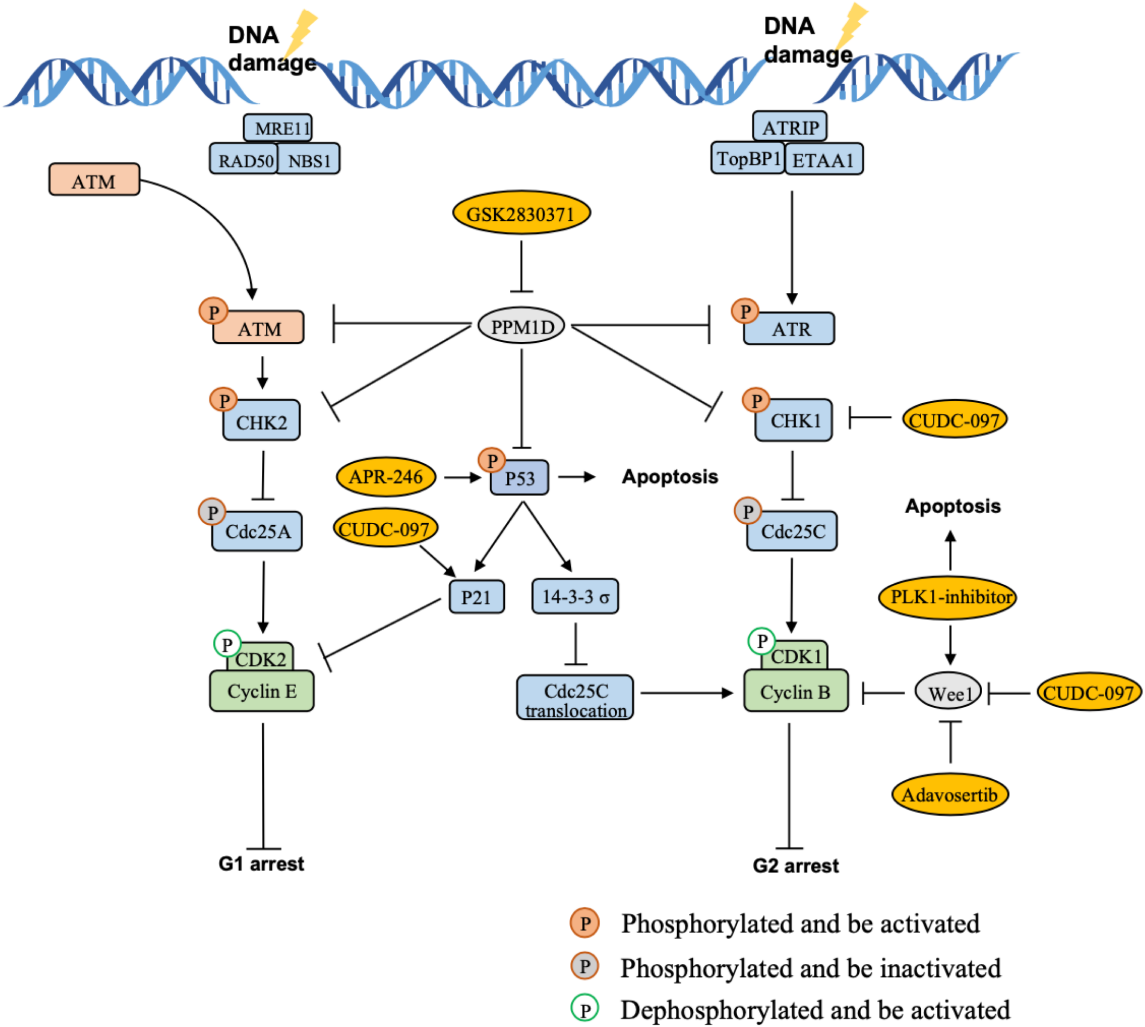
The regulation of cell cycle checkpoints in response to the irradiation‐induced DNA damage. Molecules in yellow ovals are suggested to restore the radiosensitivity in *H3* ^K27M^‐mutated DMGs.

Cyclins and cyclin dependent kinases (CDKs) are the core members regulating cell cycle through the modulation of cell cycle checkpoints.^76^ In the condition of DNA damage, recruited MRE11/RAD50/NBS1 (MRN) complex and ATR‐interacting protein (ATRIP) activate ataxia telangiectasia mutated (ATM) kinase and ataxia telangiectasia‐ and Rad3‐related protein (ATR), respectively.^74^, ^77^ Activated ATM and ATR then phosphorylate checkpoint kinases (CHKs), which can further phosphorylate the Cdc25 leading to the inactivation of CDKs, as a result, causing G1 and G2 phase arrest.^75^, ^78^, ^79^, ^80^ The function of CDKs and Cdc25 could be regulated by the modulator CDK activated kinase (CAK), Wee1, PLK1, 14–3‐3 σ, and cyclin‐dependent kinase inhibitor (CKI).^81^, ^82^, ^83^, ^84^ In addition, p53 could execute G1 phase arrest via p21 generation and accumulation^85^, ^86^, ^87^, ^88^ (Figure 2). Radiotherapy as one of the most common treatments for gliomas can damage cancer cells DNA by IR leading to mitotic catastrophe. However, glioma cells could get the radiation‐induced DNA damages repaired via G1 and G2 arrest and continue the cell cycle and proliferation to avoid cells death or senescence presenting radiotherapy resistance. Thus, targeting the checkpoints could be a promising strategy of enhancing radio‐sensitivity.^89^, ^90^

### 3.2 DNA damage and repair system

#### 3.2.1 DNA double‐strand breaks (DSBs) repair

Homologous recombination (HR) and nonhomologous end‐joining (NHEJ) are two main pathways involved in DSBs damage repairing. HR occurs in the S and G2 phases requiring a sister chromatid as template. NHEJ could participate in all cell cycle phases, predominately in G1 phase.^19^ Different from HR, NHEJ can directly ligate the broken DNA ends without resection.^20^

#### 3.2.2 Homologous recombination (HR)

**FIGURE 3:**
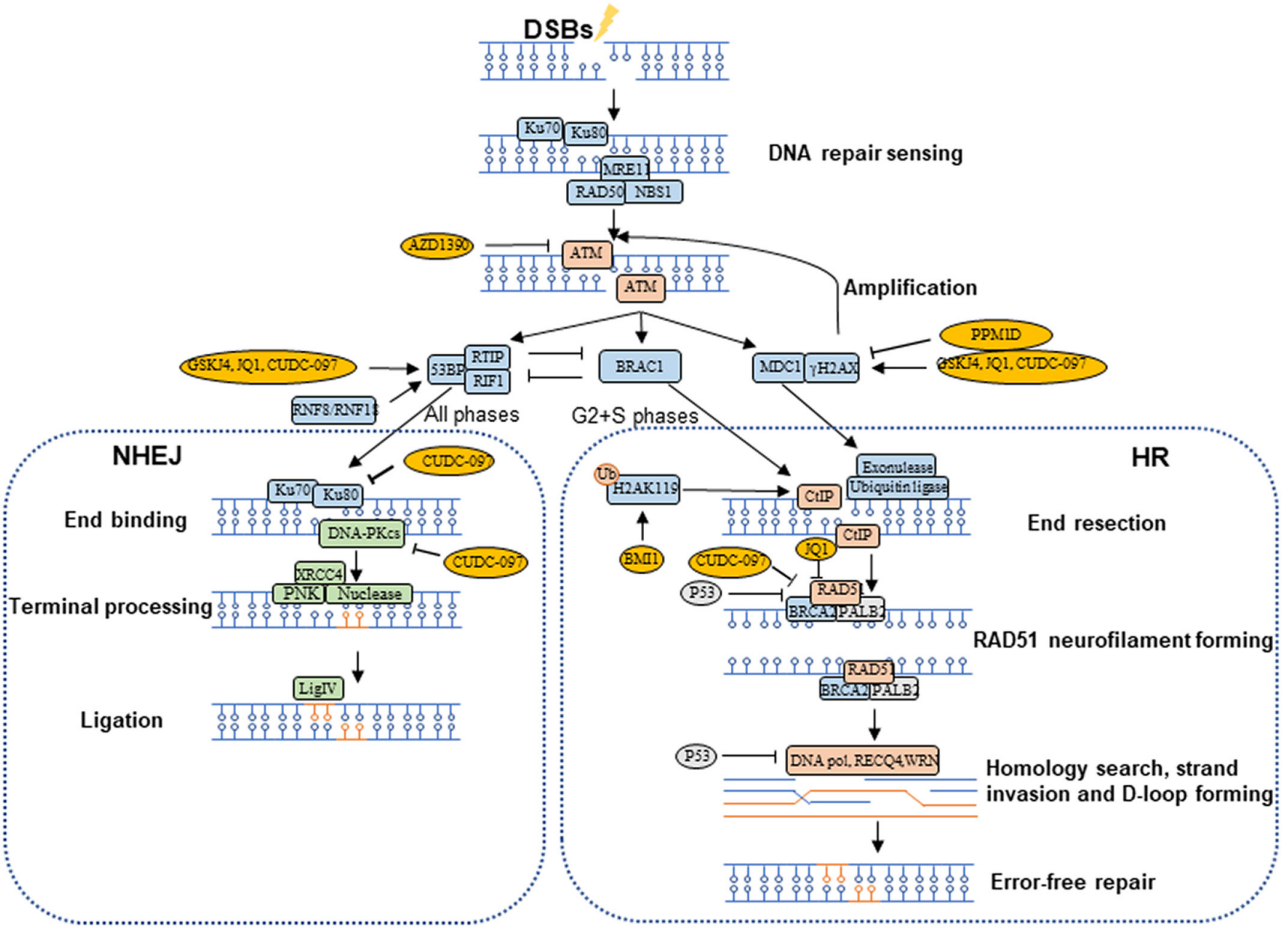
The illustration of DNA double‐strand breaks (DSBs) repair. Yellow ovals indicate the molecules potentially increasing the radiosensitivity in *H3* ^K27M^‐mutated DMGs.

HR repair can restore damaged DNA using an undisturbed sister chromatid as template during S and G2 phases. During DSBs repair process, MRN complex, as the sensor of the DNA breaks, can firstly recruit and active ATM to initiate a phosphorylation cascade of mediators, such as mediator of DNA damage checkpoint 1 (MDC1) and breast cancer type 1 susceptibility protein (BRCA1).^19^, ^91^ MDC1 is essential for signal transduction and amplification in DNA damage repair networks. For example, MDC1 can recruit a series of enzymes participating in damaged DNA ends resection including helicase, exonuclease, ubiquitin ligase, recombinase, and DNA end decorating factors.^20^, ^92^, ^93^, ^94^, ^95^ Meanwhile, MDC1 combined with γH2AX, a substrate of ATM,^96^ can further recruit additional MRN complex and ATM at the DSBs locus, resulting in the amplification of ATM signaling. BRCA1 as a scaffold protein can recruit multiple repair proteins such as CtBP‐interacting protein (CtIP) to the DNA break sites promoting the single‐strand excision of DNA ends.^73^, ^97^ CtIP then promotes an accumulation of RAD51 which can bind single‐strand DNA (ssDNA) after end resection to recognize and invade the sister chromatid forming D‐loop for the assurance of the DNA synthesis fidility.^73^, ^77^ In addition, recent studies have suggested that B lymphoma Moloney murine leukemia virus insertion region 1 (BMI1), a core member of polycomb repressive complex 1 (PRC1), could regulate the progression of HR by depositing H2A ubiquitylation at K119N (H2AK119ub) which is able to recruit CtIP and promote CtIP‐BRCA1 complex formation and consequential signaling transduction^98^ (Figure 3).

#### 3.2.3 Nonhomologous end‐joining (NHEJ)

NHEJ reaction can detect and ligate the damaged double‐strand DNA ends to restore the strand continuity without template. The Ku70/80 heterodimer firstly recognize and bind to the broken DNA ends with the recruitment and activation of DNA‐dependent protein kinase catalytic subunit (DNA‐PKcs). DNA‐PKcs then interacts with multiple enzymes including the ARTEMIS and APLF nucleases and the poly nucleotide kinase (PNK) to initiate the terminal processing by removing non‐ligatable end group at DNA damage sites.^99^ Meanwhile, DNA‐PKcs can activate x‐ray repair cross complementing protein4 (XRCC4) to keep the two broken DNA ends closely aligned.^58^ Finally, XRCC4 binds with ligase IV (LigIV) to form the XRCC4‐LigIV complex leading to the DNA ends ligation.^73^

P53 binding protein1 (53BP1) is essential to determine the initiation of either HR or NHEJ pathways. γH2AX, as a trigger of DNA damage repair, binds directly with MDC1 to activate RING finger protein 8 (RNF8)‐RNF168‐mediated ubiquitination of histone, which can induce the 53BP1 recruitment to the DNA damage site. 53BP1 further interacts with replication timing regulatory factor 1 (RIF1) and pax transactivation domain interacting protein (PTIP) to inhibit HR‐associated DNA end resection. Importantly, the function of 53BP1‐RIF1 complex and 53BP1‐PTIP complex could be negatively regulated by BRCA1^100^, ^101^, ^102^ (Figure 3).

### 3.3 DNA single‐strand break repair (SSBR)

**FIGURE 4:**
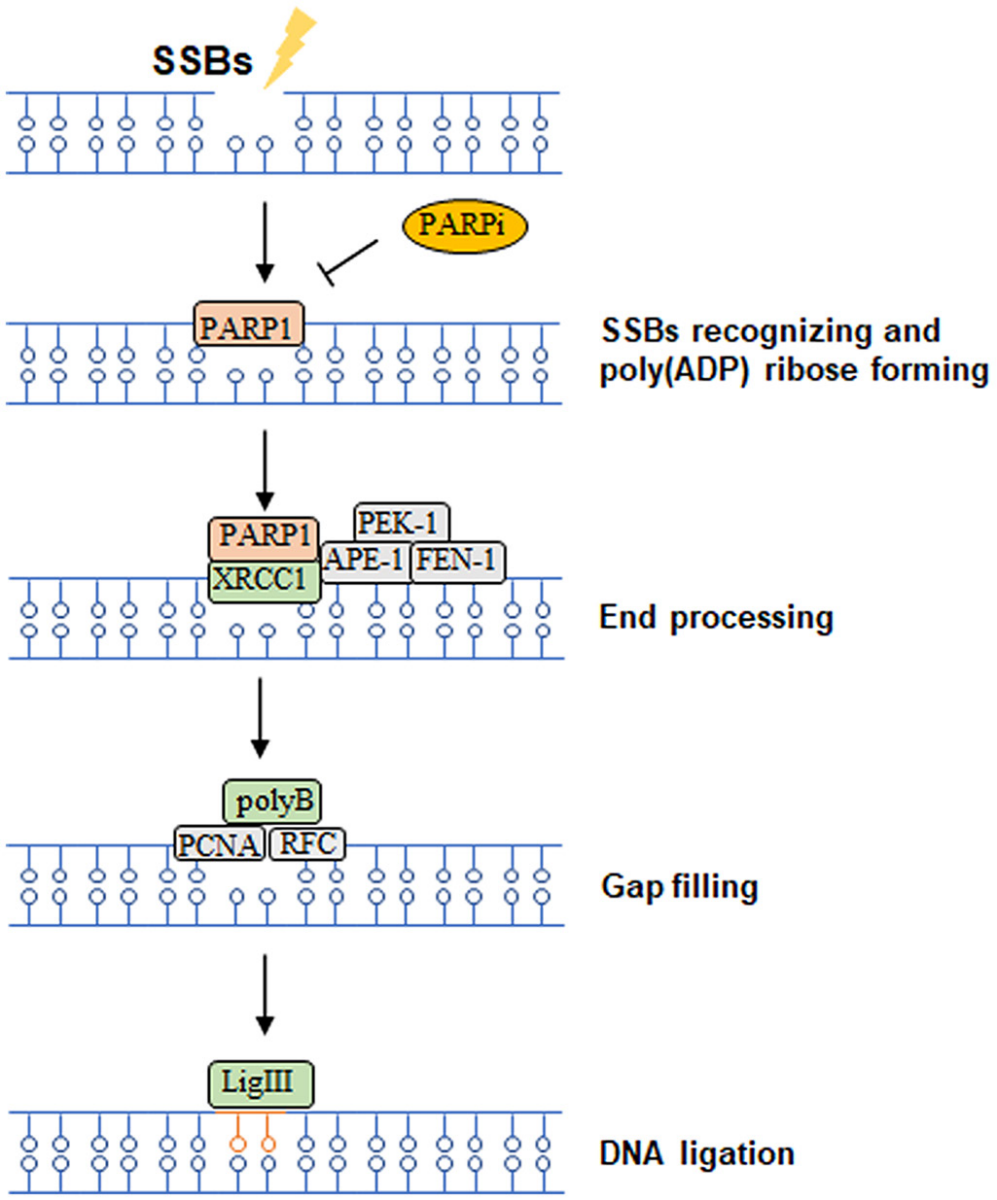
The diagram of DNA single‐strand breaks repair (SSBR) and potential targets to increase radiosensitivity in *H3* ^K27M^‐mutated DMGs (yellow oval).

SSBR responding to single‐strand breaks (SSBs) consist of SSBs recognition, end processing, gap filling and ligation. Poly (ADP‐ribose) polymerase 1 (PARP1) is the key enzyme to detect the DNA damage and induce poly‐ADP‐ribosylation (PARylation) to initiate SSBR.^103^ PARP1 then interacts with X‐ray repair cross complementing protein1 (XRCC1) resulting in the recruitment of multiple DNA repair factors such as proliferation cell nuclear antigen (PCNA), replication factor C (RFC) and DNA polymerase (DNA pol) to facilitate the end processing and gap filling. Finally, XRCC1 can bind DNA ligase III (LigIII) to execute ligation to repair the broken DNAs^104^ (Figure 4).

### 3.4 Signaling pathways regulate DNA damage repair

**FIGURE 5:**
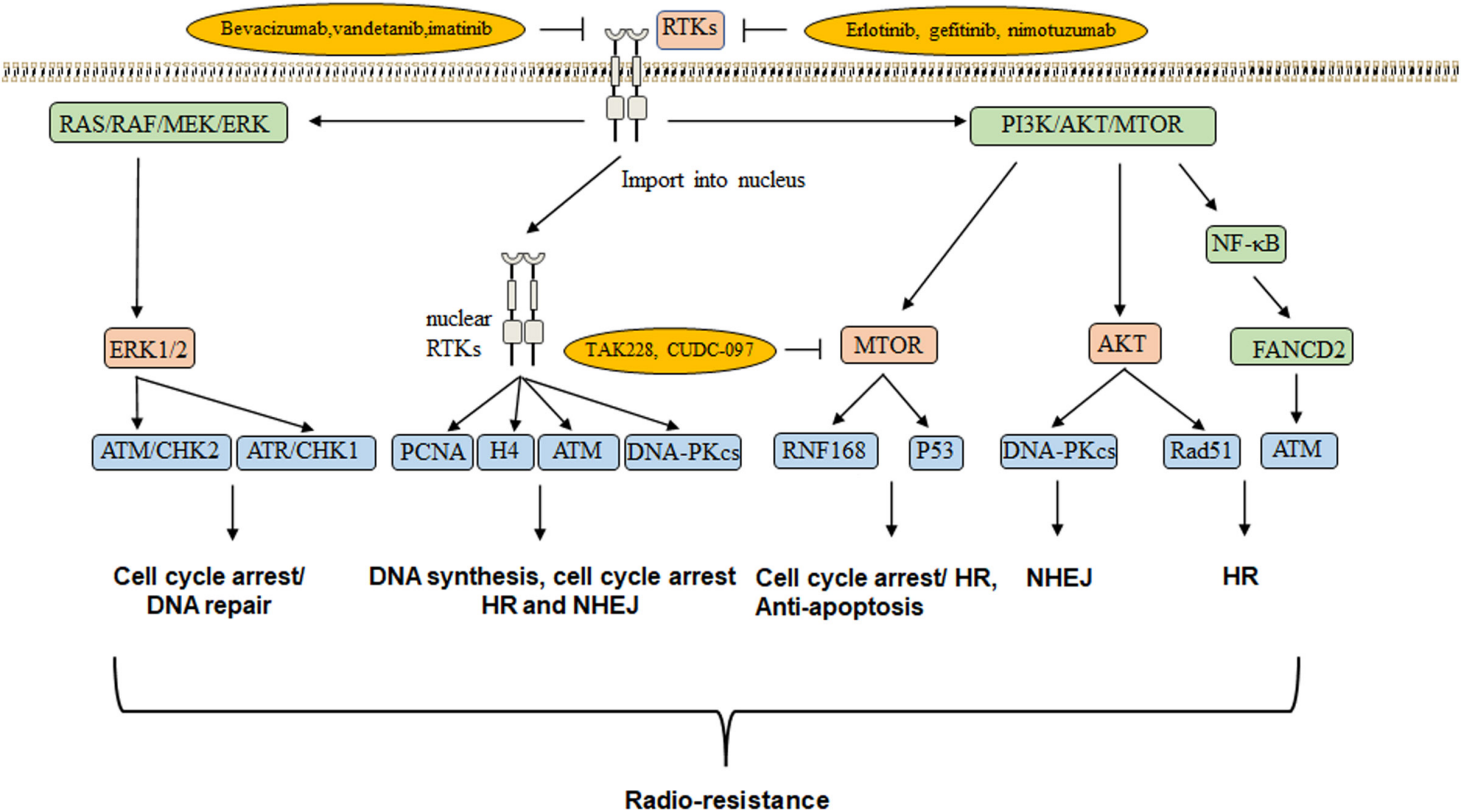
RTKs and downstream signaling associated with radio‐resistance in *H3* ^K27M^‐mutated DMGs. Yellow ovals indicate the molecules enhancing radiosensitivity in clinical and investigation.

DDR process receives regulations from multiple intra‐ and extracellular signalings.^105^ RTKs and their signaling pathways including PI3K/AKT/mTOR and MEK/ERK can regulate DDR by interacting with the components in both HR and NHEJ, such as PCNA, ATM, and DNA‐PKcs.^106^, ^107^ For example, in DSBs, AKT can activate Rad51 and DNA‐PKcs to facilitate DNA damage repair.^108^ mTOR is able to mediate DDR by regulating RNF168 ubiquitination and P53.^109^, ^110^ In addition, mTOR can increase FA complementation group D2 (FANCD2) to promote ATM‐CHKs checkpoints by activating NF‐κB pathway.^111^, ^112^ MEK/ERK pathway has been shown to interfere with cell cycle checking points and DDR by enhancing ATM and ATR activation^113^, ^114^, ^115^ (Figure 5).

## 4 RADIO‐RESISTANCE AND CURRENT RADIO‐SENSITIZATION STRATEGIES

In *H3* ^K27M^ DMGs, the genetic and epigenetic abnormalities could disrupt the cell cycle checkpoints and DDR system by directly or indirectly altering the associated regulatory signaling pathways.^9^, ^21^, ^22^, ^23^, ^72^, ^116^

Which results in the common development of radio‐resistance. Thus, current strategies of enhancing radiosensitivity of *H3* ^K27M^ DMGs mainly focus on the amendment of the *H3* ^K27M^ epigenic and genetic alterations and the aberrant cell cycle checkpoints and DDR system (Table 2), as well as the combination therapies with immunotherapy and chemotherapy.

**TABLE 2:** Potential targets to increase radiosensitivity in *H3* ^K27M^ DMGs.

Targets	Molecules	Molecular mechanisms	References
H3K27M	GSKJ4	Sustain high level of 53BP1 and γH2AX	117
BET	JQ1	Sustain high level of 53BP1 and γH2AX; decrease RAD51	118
H3K27ac	CUDC‐097	Sustain high level of 53BP1 and γH2AX; decrease RAD51, DNA PKcs, KU70/80, Weel and CHK1	119
TP53	APR‐246	Inhibit antioxidants; increase apoptosis; inhibit HR	5, 74, 89, 134, 246, 247, 248
ATM	AZD1390	Inhibit phosphorylation cascades including CHK1/2, BRAC1/2, γH2AX, MDC1and 53BP1	74, 133, 134
PPM1D	GSK2830371	Restore the activation of P53	153, 154, 155, 156, 159, 160, 161
PARP1	Niraparib	Inhibit SSBR	103, 105
Wee1	Adavosertib	Promote G2 to M transition	123, 249, 250
PLK1	BI6727	Enhance DNA damage; increase apoptosis	131, 250
CDK4/6	Ribociclib	Sustain high level of γH2AX; increase apoptosis; decrease ATM and RAD51	85, 124, 126, 128
BMI1	PTC‐209	Inhibit DNA end resection in HR	98, 135
RTKs	Nimotuzumab, Erlotinib, Gefitinib, Bevacizumab, Vandetanib, Imatinib	Inhibit mTOR and MAPK pathways	9, 164, 165, 251, 252
PI3K/AKT/MTOR	TAK228, CUDC‐097	Induce sustained DNA damage and apoptosis; reduce DDR	119, 176

### 4.1 Targeting epigenetic alterations

A global reduction of a H3K27me3 and increased H3K27ac are two hallmarks in *H3* ^K27M^ DMGs.^9^, ^21^ Recent studies suggest that targeting the epigenetic abnormalities could increase the responses of K27M tumors to radiotherapy. The inhibitor of H3K27 demethylase JMJD3, BET bromodomain protein (BRD) and a dual inhibition of histone deacetylases (HDAC) and PI3K have been demonstrated to sensitize the radiotherapy via enhancing radiation damage and inhibiting DNA damage repair.^117^, ^118^, ^119^ A recent study showed targeting EZH2 had a tumor suppressor function in DMG tumor bearing mice, which could potentially enhance the radiotherapy.^120^

### 4.2 Targeting cell cycle checkpoints and DDR system

Cell cycle checkpoints and DDR intertwined signaling networks can arrest cell cycles, identify, and repair DNA damages induced by radiation. Targeting the components of cell cycle checkpoints and DDR is one of the main strategies to rescue radio‐resistance. In *H3* ^K27M^ DMGs, the abnormalities of G1/S checkpoint regulators such as CDK4/6 and cyclinD and G2/S regulators such as Wee1, PLK1 were commonly detected.^121^, ^122^, ^123^, ^124^ Although the block of CDK4/6 could result in G1 arrest which potentially decrease the sensitivity of radiotherapy, CDK4/6 inhibition alone or following radiotherapy have been suggested to significantly reduce tumor growth and improve survival in DIPGs,^125^, ^126^, ^127^ which is possibly due to the increased sensitivity to radiation of G1 arrested tumor cells, and failing in the requirement of sustained DNA damage during cell cycle stocked.^89^, ^128^ Wee1 acting as a regulator of G2 cycle could repair irradiation‐induced DNA damage by suppressing Cdc25 and CDK1 activation.^129^ The Wee1 inhibitor adavosertib has been demonstrated to promote the radiation responses in DIPGs though the suppression of G2 cycle arrest.^130^ Although PLK1 negatively regulates Wee1 activation, its inhibition induces accelerated apoptosis and enhanced DNA damage of DIPG cells during radiotherapy.^131^ Besides, the inhibition of Aurora kinase A (AURKA), as a key cell cycle kinase in M phase, could block cell cycle progression synergistically combined with *PLK1* inhibitor in DMG.^132^ ATM as a key modulator in the networks of cell cycle checkpoints and DDR initiates a series phosphorylation cascades including CHK1/2, BRAC1/2, MDC1, γ‐H2AX and 53BP1^74^, ^133^ ATM deletion is proved to enhance tumor radiosensitivity in p53‐deficient DIPG,^134^ and its inhibitor AZD1390 is currently involved in a clinical trial of the brain cancer patients underwent radiation therapy (NCT03423628). In addition, the enzymes such as BMI1 and PARP1 are hyperactive in DIPG.^135^, ^136^ The inhibition of BMI1 could sensitize the DIPG cells response to radiotherapy via inhibiting damaged DNA ends resection in HR.^135^ PARP1 is important for the recognition of SSBs and recruitment of XRCC1.^105^ The inactivation of PARP1 exhibits enhanced radiosensitivity in DIPGs.^137^, ^138^ However, a phase I/II study of veliparib combined with chemoradiotherapy in younger patients with newly diagnosed DIPG could not improve the the survial.^139^

### 4.3 Targeting P53 and PPM1D

P53 participates in the G1/S phase arrest via Retinoblastoma (RB) pathway and contributes to the G2 arrest via 14‐3‐3 σ induced sequestration of Cdc25/CDK2 complexes in the cytoplasm.^140^ TP53 mutation frequently occurs in *H3* ^K27M^ DMGs.^141^, ^142^ The lost‐of‐function of P53 in *H3* ^K27M^ DMGs can decrease Bax‐induced apoptosis and promote HR process in DDR resulting in a general poor response to radiotherapy.^134^, ^143^, ^144^, ^145^, ^146^, ^147^, ^148^, ^149^, ^150^, ^151^ Thus, restoring P53 could be a potential treatment of mutant TP53‐mutated *H3* ^K27M^ DMGs. A recent study suggests that reactivation of P53 combined with *H3*K27M demethylase inhibitor augments the therapeutic efficacy of irradiation in *H3* ^K27M^ DIPGs.^152^

PPM1D amplification has been identified in DMGs,^153^, ^154^, ^155^, ^156^ which present resistance to radiotherapy. PPM1D can dephosphorylate and inactivate P53 and a series of key enzymes in cell cycle checkpoints and DDR, including ATM, ATR, Chk1/2 and γH2AX, leading to cell cycle escape and failing in DDR.^155^, ^157^, ^158^, ^159^, ^160^, ^161^ Recent studies show that inhibition of gain‐of‐function of mutated PPM1D can increase radiosensitivity of DIPG tumors through restoring the activation of p53,^156^, ^162^ and targeting PPM1D would sensitize DIPG cells to the *PARP* inhibitor due to dual suppression of HR and SSBR.^163^

### 4.4 Targeting RTKs and downstream signaling pathways

RTKs and their downstream pathways regulate the cell cycle checkpoints and DDR process involving in the response of tumor cells to radiotherapy and radio‐resistance in DMGs.^22^, ^23^, ^72^ Although the clinical benefit of targeting RTKs such as EGFR and vascular endothelial growth factor receptor (VEGFR) in combination with radiotherapy are still not conclusive in *H3* ^K27M^ DMGs or DIPGs.^9^, ^56^, ^164^, ^165^, ^166^, ^167^, ^168^, ^169^, ^170^ The inhibition of their downstream PI3K/AKT/mTOR and MEK/ERK pathways can significantly sensitize glioma cells to radiotherapy exhibiting increased cell apoptosis and reduced cancer stemness.^171^, ^172^, ^173^, ^174^, ^175^, ^176^, ^177^ The mTOR inhibitor TAK228 has been suggested to induce cell apoptosis and enhance radiosensitivity in DIPG.^176^ In addition, combined targeting strategy is under estimation. A study of dual inhibition of PI3K and HDACs in DIPGs shows an increased radiosensitivity though abrogating NFκB and Forkhead box M1 (FOXM1) mediated in DNA damage responses.^119^, ^178^, ^179^ FOXM1 is a transcription factor regulating the expression of cell cycle genes essential for DNA replication and mitosis, and DNA damage repair.^178^

### 4.5 Targeting cancer‐stemness and EMT

Accumulating evidence suggests that cancer stem cells (CSCs) in DMGs impact the sensitivity of radiotherapies.^177^, ^180^, ^181^ CSCs are a small subpopulation of cells within tumors with capabilities of self‐renewal, differentiation, and tumorigenicity. The stemness of CSCs regulated by intrinsic factors such as cell survival and self‐renewal pathways^182^, ^183^, ^184^, ^185^, ^186^, ^187^, ^188^, ^189^ and extrinsic causes such as hypoxia microenvironment^190^, ^191^, ^192^, ^193^, ^194^ allow to activate cell cycle check points and DDR to decrease the DNA damage from radiation and develop radio‐resistance.^182^ Notch pathway is considered to be an essential self‐renewal signaling to maintain CSCs stemness in *H3* ^K27M^ gliomas, while its inhibitor, MRK003, presents an enhanced irradiation‐induced apoptosis of tumor cells.^195^ Recently, targeting mitochondrial metabolism and tumor hypoxia microenvironment are suggested to effectively ameliorate radio‐resistance in CSCs in high‐grade gliomas.^196^

EMT is commonly involved in tumor initiation, invasion and metastasis in a broad set of cancers, which presents a transition of epithelial cells to mesenchymal cells with the lost function of cell–cell junctions and cell polarity.^197^, ^198^, ^199^, ^200^, ^201^, ^202^, ^203^ EMT requires a robust reprogramming of gene expression which is associated with maintenance of cancer stemness leading chemo‐ and radio‐resistance.^204^, ^205^, ^206^, ^207^ EMT programs are regulated by multiple pathways including JAK/STAT pathway, TGF‐β/SMAD, NF‐κB, PI3K/AKT signaling, and Notch signaling,^208^, ^209^, ^210^ which offer the potential targets to enhance the sensitivity of radiotherapy. Recently, several preclinical studies suggested that the inhibition of STAT3 and Notch pathways significantly increase the therapeutic response to radiotherapy in DIPGs.^211^, ^212^ In addition, sonic hedgehog (SHH) pathway is considered as an EMT‐promoting signaling promoting tumor cell differentiation and invasion in gliomas.^213^ Its inhibition is shown to effectively arrest tumor growth in various types of cancers.^214^, ^215^, ^216^, ^217^ A clinical trial about SHH pathway inhibitor, Vismodegib, in the refractory pontine glioma patients was on going.^218^ Pursuing further studies in DMGs would be appreciated.

### 4.6 Other potential targets

Recent clinical trials suggested that ONC201, a dopamine receptor D2 (DRD2) inhibitor, combined with radiotherapy can significantly improve the prognosis of *H3* ^K27M^ DMGs.^219^, ^220^ Although ONC201 was reported to activate ATF4/CHOP‐mediated integrated stress response and inhibit Akt/ERK signaling leading to the apoptosis in cancer cells, the mechanism of radio‐sensitization is still unknown. Further studies are be appreciated. BCL2 inhibitor venetoclax could reduce BCL2‐BIM association and increase ROS resulting in tumor apoptosis. Radiotherapy combined with venetoclax was reported to suppress tumor growth in DMGs.^221^ Future investigation in radio‐sensitization in *H3* ^K27M^ DMGs treatment are encouraging.

### 4.7 Combination with immunotherapy and chemotherapy

*H3* ^K27M^ DMGs are considered as “immune cold” tumors with reduced residency of immune cells and lack of factors or chemokines recruiting immune cells.^222^, ^223^, ^224^ Radiation can stimulate the release of tumor antigens and pro‐inflammatory factors and immune cells infiltration to enhance immune responses in the tumor microenvironment.^225^, ^226^, ^227^ Conversely, radiosensitivity can be affected by the activation of immune cells such as macrophages and fibroblasts in tumor and tumor microenvironment.^228^ Chimeric antigen receptor (CAR)‐T cell therapy targets tumor via endowing T cells with the specific tumoral antigens,^229^ which have been used in the treatment of *H3* ^K27M^ DMGs. The newest clinical trials showed that GD2 and B7‐H3 CAR‐T cells therapies following standard radiotherapy are beneficial and safe for *H3* ^K27M^ DMGs or DIPGs.^230^, ^231^ Additionally, a latest randomized clinical trial demonstrated the patients undergoing concurrent radio‐chemotherapy with interferon α had a longer survival time than those without interferon α in high‐grade gliomas.^232^ The oncolytic virus Delta‐24‐RGD is recently reported to elicit an elevated sensitivity of *H3* ^K27M^ DMGs to radiotherapy by increasing the trafficking of CD4^+^ and CD8^+^ cells to the tumor niche, and the downregulation of kinase activities in DDR system.^233^, ^234^ Another oncolytic virus DNX‐2401 followed by radiotherapy can reduce and stabilize tumor size in pediatric DIPGs clinical trial.^235^ Some other immunotherapeutic options also include vaccines and checkpoint blockade; however, it was still in clinal trial phase.^218^ To date, the interacted mechanisms between radiotherapy and immunotherapy have not completely unknown in *H3* ^K27M^ DMGs. Further studies would be warranted to explore the synergetic mechanism on radiation combined with immunotherapy.

As for chemotherapy, the combination of temozolomide (TMZ) and radiotherapy is recommended in *H3* ^K27M^ DMGs. However, the clinical of outcomes are not satisfied due to the unmethylation of the O^6^‐methylguanine‐DNA methyltransferase (MGMT) associated facilitation of DNA damage repair, and relatively intact blood–brain barrier (BBB).^13^, ^14^, ^236^, ^237^, ^238^, ^239^, ^240^, ^241^, ^242^ Recently, the development of nanotechnologies allows to reformulate TMZ to enhance therapeutic efficiency with the evasion of MGMT enzyme.^243^ In addition, pulsed ultrasound and convection‐enhanced delivery (CED) have been applied to temporally disrupt the BBB and increase the concentration of drug delivery in tumor area, as a result, to enhance the sensitivity of chemotherapies and radiotherapy.^244^, ^245^

## 5 CONCLUSIONS

The clinical treatment of *H3* ^K27M^ DMGs remains challenging. Beyond maximal safe tumor resection, adjuvant radiotherapy is reliable treatment to improve patients' outcomes. However, the genetic and epigenetic alterations and signaling dysregulation participating in cell cycle checkpoints and DDR in *H3* ^K27M^ DMGs frequently associates with radio‐resistance resulting in unsatisfied prognoses. Recent advances in these mechanisms have tremendously improved our understandings of radio‐resistance in *H3* ^K27M^ DMGs and allowed to develop the potential targets to enhance the sensitivity to radiotherapy. Further clinical trials would be appreciated to precede successful radiotherapy‐enhancing interventions and eventually improve the survival of *H3* ^K27M^ DMGs.

## AUTHOR CONTRIBUTIONS

Chao Liu and Longbo Zhang searched the literature and wrote the manuscript; all authors contribute to reviewing and editing.

## FUNDING INFORMATION

This work was supported by the National Natural Science Foundation of China (grant No. 82171171 (L.Z.)), and the Natural Science Foundation of Human Province (grant No. 2018JJ3856 (C.L.) and grant No.2018JJ3824 (X.G.)).

## CONFLICT OF INTEREST STATEMENT

The authors declare no conflict of interest.

## INSTITUTIONAL REVIEW BOARD STATEMENT

Not applicable.

## INFORMED CONSENT STATEMENT

Not applicable.

## Data Availability

No new data were created.
